# Impact of a Soil Cyanobacteria Consortium-Based Bioinoculant on Tomato Growth, Yield, and Fruit Quality

**DOI:** 10.3390/plants14132034

**Published:** 2025-07-02

**Authors:** Zineb Hakkoum, Farah Minaoui, Zakaria Tazart, Amer Chabili, Mountasser Douma, Khadija Mouhri, Mohammed Loudiki

**Affiliations:** 1 Laboratory of Water Sciences, Microbial Biotechnologies, and Sustainability of Natural Resources, Department of Biology, Faculty of Sciences Semlalia, Cadi Ayyad University, UCA, Bd Prince Moulay Abdellah, Marrakesh 40000, Morocco; zineb.hakkoum@gmail.com (Z.H.); minaoui.farah@gmail.com (F.M.); zakaria.tazart@um6p.ma (Z.T.); chabiliamer@gmail.com (A.C.); douma_mountasser@yahoo.fr (M.D.); mouhri@uca.ac.ma (K.M.); 2 Higher Institute of Nursing and Health Technology Professions, Laayoune 70000, Morocco; 3 AgroBioSciences, Plant Stress Physiology Laboratory, Mohammed VI Polytechnic University, Benguerir 43150, Morocco

**Keywords:** soil cyanobacteria, consortium, biofertilizer, soil fertility, tomato growth, fruit quality

## Abstract

Cyanobacteria-based bioinoculants represent a sustainable solution for enhancing soil fertility and crop productivity. This research assessed the biofertilizing potential of two indigenous nitrogen-fixing cyanobacteria strains (*Nostoc punctiforme* Har. and *Anabaena cylindrica* Lemmerm.) on tomato growth and yield. A greenhouse experiment was conducted to study their effects on soil properties, plant growth and physiology, and fruit yield/quality. The strains were applied individually, as a consortium, or combined with organic or mineral fertilizers at half the standard dose (50%). All bioinoculants improved soil fertility, plant growth, and fruit yield/quality compared to the control. The most significant improvement was observed in the consortium amended with 50% of conventional fertilizer (compost or NPK), compared with individual strains. Correlation analysis revealed strong positive associations between photosynthetic pigments, plant productivity, and fruit biochemical traits, indicating coordinated physiological responses under the applied treatments. The results demonstrated that the consortium of diazotrophic terrestrial cyanobacteria possesses tomato biofertilizer properties that can be efficiently used in crop production. These findings suggest that such formulations offer a cost-effective approach to tomato cultivation and present a sustainable alternative for integrated and optimized fertilizer management.

## 1. Introduction

Contemporary agricultural practices face significant challenges due to the overreliance on synthetic agrochemicals to boost crop yields. These challenges include soil degradation, environmental pollution, and a decline in microbial biodiversity [1,2,3]. As an eco-friendly alternative, biofertilizers such as mycorrhizae, rhizobacteria, symbiotic nitrogen-fixing bacteria, microalgae, and cyanobacteria offer sustainable solutions to these issues by enhancing nutrient cycling, improving soil health, and reducing the need for chemical inputs. These biological agents confer numerous benefits to plants, including improved nutrient acquisition through nitrogen fixation and phosphate solubilization, production of growth-promoting phytohormones (e.g., auxins and cytokinins), induction of systemic resistance to pathogens and drought stress, and enrichment of beneficial rhizosphere microbiota; collectively, these functions contribute to a reduced dependency on synthetic chemical fertilizers [3,4,5,6,7,8,9,10,11]. Cyanobacterial biofertilizers are gaining increasing attention due to their numerous advantages, such as high photosynthetic efficiency and the production of agriculturally beneficial metabolites and plant growth-promoting substances such as phytohormones, polysaccharides, and amino acids. Additionally, they enhance and modulate soil microbial activity, improve soil quality and fertility, and increase crop productivity [4,7,12,13].

Over the past decade, numerous studies have recommended the use of cyanobacteria or their by-products as biofertilizers not only for rice but also for many other crops, including wheat (*Triticum aestivum* L.), cantaloupe (*Cucumis melo* L.), radish (*Raphanus sativus* L.), tomato (*Solanum lycopersicum* L.), pepper (*Capsicum annuum* L.), soybean (*Glycine max* (L.), common bean (*Phaseolus vulgaris* L.), oat (*Avena sativa* L.), maize (*Zea mays* L.), lettuce (*Lactuca sativa* L.), and cotton (*Gossypium hirsutum* L.) [4,13,14,15,16,17,18,19]. Cyanobacteria-based biofertilizers have been shown to improve plant growth, fruit quality, nutritional characteristics, and seed yield [14,16,20,21,22]. Indeed, cyanobacteria are now considered one of the most important biofertilizers in agriculture, as they represent a renewable source of biomass rich in organic substances and secondary metabolites that can be mineralized by soil microflora [7,18,23]. Specific physiological and metabolic traits of cyanobacteria, such as their ability to retain water, fix atmospheric nitrogen (N_2_), exhibit a short generation time, and survive in harsh environments, make them an effective biofertilization source for improving soil physicochemical properties and fertility [18,23,24,25].

Numerous studies have demonstrated that cyanobacteria, particularly those *nitrogen-fixing* in the genera *Anabaena* and *Nostoc*, can serve as effective biofertilizers [7,13,18,26]. These genera are considered a highly promising source of beneficial strains [7,13,18,26]. Research has highlighted the capability of indigenous *Nostoc* and *Anabaena* strains to enhance soil fertility, increase soil microbial biomass, promote plant growth, boost available nitrogen levels, improve seed germination, and increase crop yields [7,16,27,28]. However, most studies have focused on the biofertilizing effect of single strains, often isolated from aquatic environments [1,18]. In contrast, fewer studies have examined the biofertilizer potential of soil-indigenous cyanobacterial consortia applied directly to soils through various formulation approaches [4,12,29]. Soil inoculation with live and fresh cyanobacterial biomass is generally advantageous, as it provides continuous nutrient sequestration throughout plant growth phases. Additionally, it offers benefits such as supplying organic matter, reducing soil erosion, preventing nutrient leaching, and maintaining soil structure and fertility [13,19,30]. Furthermore, one of the most significant advantages of cyanobacteria-based biofertilizers is their ability to enhance soil organic carbon, a benefit unattainable with chemical fertilizers [3,7,10,28,31].

The deployment of live cyanobacterial consortia, which exhibit diverse beneficial traits and synergistic effects, is now recognized as an effective strategy for improving biofertilizer efficacy, particularly in enhancing nutrient uptake, promoting plant growth, and improving soil structure and fertility [3,4,12,32]. Furthermore, recent studies have demonstrated that cyanobacterial consortia exhibit greater potential than monocultures in producing metabolites and serving as biofertilizers in agriculture [4,32]. Experimental studies on the association between N_2_-fixing cyanobacteria and agronomically important crops have yielded promising results [4,12,26,27]. Accordingly, the present study aimed to evaluate the biofertilizing effect of inoculating two native soil cyanobacteria, *Nostoc punctiforme* and *Anabaena cylindrica*, as a consortium applied alone or in combination with compost or mineral (NPK) fertilizers on tomato plant growth, fruit yield, and soil fertility. This study should test the hypothesis that tomato growth, crop yield, and soil fertility may be more effectively improved using a cyanobacterial consortium than individual strains while reducing reliance on conventional fertilizers. Specifically, this research examines the impact of fresh cyanobacterial biomass inoculation on soil physicochemical properties; tomato plant growth and yield; physiological, biochemical, and nutritional traits; and tomato fruit quality.

## 2. Results

### 2.1. Growth and Physiological Properties of Cyanobacterial Strains

**Figure 1:**
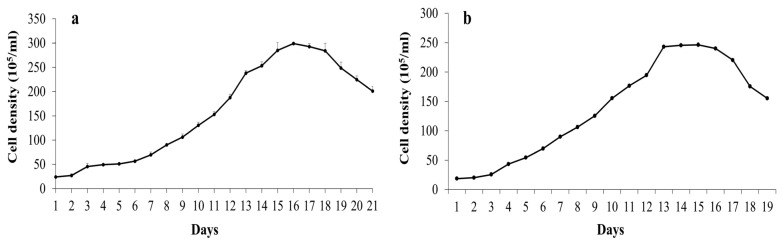
Growth kinetics of *Anabaena cylindrica* (**a**) and *Nostoc punctiforme* (**b**) in Z8 medium expressed as cell density (cells × 10^5^/mL). Data represent the mean ± SE of three replicates (*n* = 3).

The growth kinetics of *A. cylindrica* and *N. punctiforme*, expressed as cell number over a 21-day cultivation period, are shown in Figure 1. *A. cylindrica* displayed exponential growth between days 6 and 15, entering the stationary phase by day 15 (Figure 1a). In comparison, *N. punctiforme* demonstrated faster growth initiation, with the exponential phase occurring from days 3 to 13 and reaching the stationary phase by day 13 (Figure 1b). Under suboptimal culture conditions, significant differences were observed in the growth profiles and biomass of the two strains. The specific growth rate of *A. cylindrica* (µ = 0.2 day^−1^) was significantly higher than that of *N. punctiforme* (µ = 0.17 day^−1^) (Table 1). Furthermore, *A. cylindrica* demonstrated higher biomass production and chlorophyll content compared to *N. punctiforme*.

The evaluation of specific physiological traits revealed that both heterocystous filamentous cyanobacteria possess significant potential for enhancing soil fertility and promoting plant growth. Both strains exhibited substantial abilities to fix atmospheric N_2_, solubilize tricalcium phosphate, and produce extracellular polymeric substances (EPS) and the phytohormone indole-3-acetic acid (IAA) (Table 1). The *A. cylindrica* strain showed significantly higher capabilities in synthesizing IAA (12.74 ± 0.46 μg mL^−1^) and EPS (42.73 ± 1.40 mg g^−1^ DW) compared to *N. punctiforme*.

### 2.2. Cyanobacterial Biochemical Composition

The biochemical composition of the two cyanobacterial strains biomass indicated significant differences (*p* < 0.05), while pH and conductivity remained statistically similar (one-way ANOVA, *p* > 0.05). *A. cylindrica* exhibited significantly higher contents (*p* < 0.05) of phycocyanin (122.5 ± 3.1 mg L^−1^), polyphenols (102.9 ± 0.5 µg gallic acid equivalent mL^−1^), flavonoids (63.5 ± 0.4 µg catechin equivalent mL^−1^), carbohydrates (32.1 ± 1.7 mg g^−1^ DW), total organic carbon (22.22 ± 0.00%), and total Kjeldahl nitrogen (2.31 ± 0.02%). In contrast, *N. punctiforme* contained significantly higher levels (*p* < 0.05) of phycoerythrin (55.4 ± 4.0 mg L^−1^), proteins (85.1 ± 5.3 mg g^−1^ DW), Ca^2+^ (0.33 ± 0.02%), and Na^+^ (0.13 ± 0.01%). No significant differences were observed between the two strains in terms of phosphorus and allophycocyanin contents (Table 2).

The microcystin analysis revealed that neither of the two cyanobacterial strains tested positive in the PP1 toxicity assay, even at very high concentrations. Additionally, the antagonism assays showed no clear inhibition zone between the selected strains, confirming their compatibility.

### 2.3. Effect of Cyanobacterial Bioinoculants on Soil Fertility

The ANOVA results revealed that all cyanobacterial treatments enhanced the soil’s physicochemical properties in comparison with the non-inoculated control (*p* < 0.05) (Table 3). The most significant improvements were observed in soils inoculated with the *A. cylindrica* and *N. punctiforme* consortium, either alone or combined with 50% of conventional fertilizer (compost or NPK), compared to soils treated with individual strains. These cyanobacterial bioinoculants significantly reduced soil pH (*p* < 0.05) while increasing conductivity, TOC, TKN, available P, and micronutrients (K^+^, Na^+^, and Ca^2+^) (*p* < 0.05). The highest TOC content (1.75 ± 0.03%), available P (302 ± 37 mg kg^−1^), Na^+^ (345.8 ± 3.7 mg kg^−1^), and Ca^2+^ (1373 ± 13 mg kg^−1^) were observed in soil treated with the *N. punctiforme* and *A. cylindrica* consortium combined with 50% compost. These values represent increases of 150%, 105.4%, 39.70%, and 25.36%, respectively, compared to the control. Conversely, the highest TKN (363 ± 11 mg kg^−1^) and K^+^ (1282 ± 34 mg kg^−1^) contents were observed in soil inoculated with the *A. cylindrica* and *N. punctiforme* consortium combined with 50% NPK, corresponding to increases of 77.78% and 36.52%, respectively, compared to the control. Notably, in these two combined treatments, the improvements were significantly comparable to the positive controls (C^+^_NPK_ and C^+^_Comp_), which contained a full dose of NPK or organic compost (Table 3). Moreover, cyanobacterial treatments showed distinct advantages, as the concentration of total nitrogen was higher in soils inoculated with cyanobacterial consortium, amended with 50% compost, than in those treated with compost alone (100%). While compost (full dose) appears to be more effective for some soil parameters, the results demonstrated that the amended cyanobacterial consortium also showed beneficial effects in improving soil nitrogen fertility.

### 2.4. Effects of Cyanobacterial Bioinoculants on Tomato Growth

**Figure 2:**
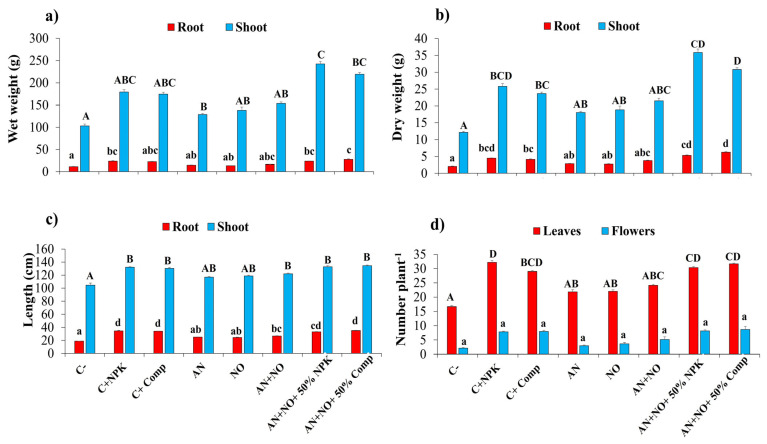
Influence of cyanobacterial treatments on growth parameters of tomato plants. Fresh weight (**a**); dry weight (**b**); root and shoot length (**c**); number of leaves and flowers/plant (**d**). C^−^: negative control; C^+^_NPK_: positive control, full dose of NPK (134 kg N/ha, 56 kg P/ha, and 276 kg K/ha); C^+^_Comp_: positive control, full dose of organic compost (250 g/kg); AN: fresh biomass of *Anabaena cylindrica*; NO: fresh biomass of *Nostoc punctiforme*; AN + NO: consortium of *Anabaena cylindrica* + *Nostoc punctiforme*; AN + NO + 50% NPK: consortium of *Anabaena cylindrica* + *Nostoc punctiforme* + 50% NPK; AN + NO + 50% Comp: consortium of *Anabaena cylindrica* + *Nostoc punctiforme* + 50% compost. Data are presented as mean ± SE (*n* = 6); means with different lowercase or uppercase letters are statistically different according to Tukey’s HSD test (*p* < 0.05).

The results demonstrated that all treatments increased the growth parameters of tomato plants compared to the untreated plants (Figure 2). Soil inoculation with a cyanobacterial consortium (*A. cylindrica* and *N. punctiforme*, AN + NO) stimulated tomato growth more effectively than individual cyanobacterial strains and the negative control. The highest increases in shoot fresh weight (135.55%) and shoot dry weight (195.14%) were recorded in plants treated with the consortium combined with half the recommended dose of NPK (AN + NO + 50% NPK) (Figure 2a,b). Meanwhile, the highest increases in root length (86.93%), shoot length (28.34%), number of leaves (89.98%), and number of flowers per plant (299.53%) were observed in plants treated with the consortium combined with half the recommended dose of compost (AN + NO + 50% Comp) (Figure 2c,d). Interestingly, for both combined treatments, the root and shoot weights and lengths were significantly higher (*p* < 0.05) than those of the positive controls containing full doses of NPK (C^+^_NPK_) or organic compost (C^+^_Comp_) (Figure 2).

### 2.5. Effect of Cyanobacterial Bioinoculants on Fruit Agronomic Parameters and Tomato Yield

**Figure 3:**
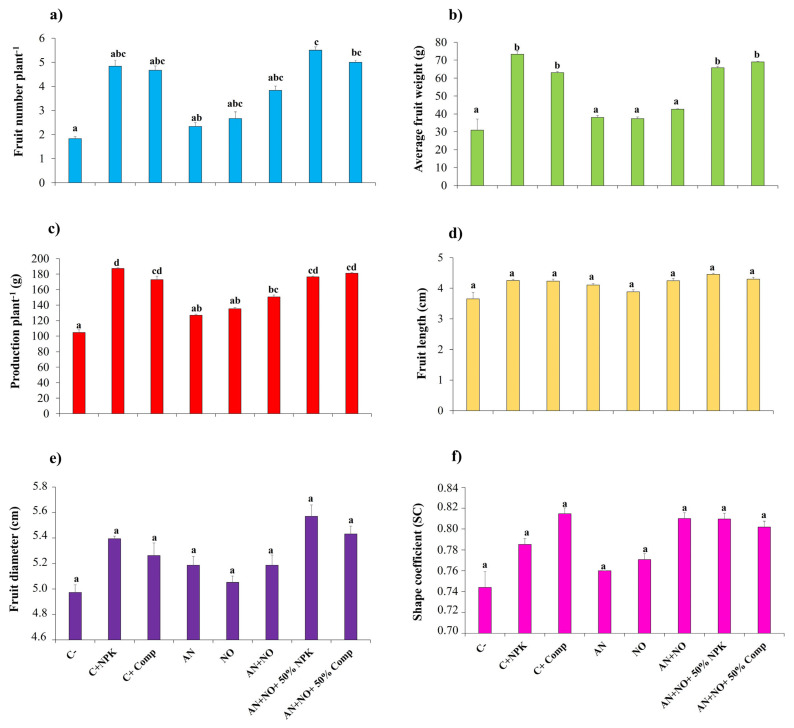
Agronomic parameters of tomato fruits under different cyanobacterial treatments. (**a**) Fruit per plant^−1^; (**b**) average fruit weight; (**c**) production per plant ^−1^; (**d**) fruit length (cm); (**e**) fruit diameter (cm); (**f**) shape coefficient (SC). C^−^: negative control; C^+^_NPK_: positive control, full dose of NPK (134 kg N/ha, 56 kg P/ha, and 276 kg K/ha); C^+^_Comp_: positive control, full dose of organic compost (250 g/kg); AN: fresh biomass of *Anabaena cylindrica*; NO: fresh biomass of *Nostoc punctiforme*; AN + NO: consortium of *Anabaena cylindrica* + *Nostoc punctiforme*; AN + NO + 50% NPK: consortium of *Anabaena cylindrica* + *Nostoc punctiforme* + 50% NPK; AN + NO + 50% Comp: consortium of *Anabaena cylindrica* + *Nostoc punctiforme* + 50% compost. Data are presented as mean ± SE (*n* = 6); means with different lowercase letters are statistically different according to Tukey’s HSD test (*p* < 0.05).

The results indicated that applying fresh biomass of cyanobacteria alone (*A. cylindrica*, AN, or *N. punctiforme*, NO) increased the number and productivity of fruits per plant compared to the negative control (Figure 3). This increase was even more pronounced in plants treated with the consortium of both cyanobacteria (AN + NO). However, these treatments did not show significant differences compared to the negative control in terms of fruit number, average fruit weight, fruit diameter, and shape coefficient. The visual differences suggested by standard errors were not supported by Tukey post hoc test results (*p* < 0.05). Conversely, inoculating plants with the cyanobacterial consortium combined with either chemical (AN + NO + 50% NPK) or organic (AN + NO + 50% Comp) fertilizers significantly increased (*p* < 0.05) both fruit production per plant and average fruit weight compared to the negative control (C^−^) and treatments with cyanobacterial bioinoculants alone (AN or NO) (Figure 3). Interestingly, this increase was comparable to that of the positive controls (C^+^_NPK_ and C^+^_Comp_), with no significant differences observed, particularly in fruit number, average fruit weight, and fruit production per plant. Specifically, the highest number of fruits per plant was recorded in the treatments combining the cyanobacterial consortium with either 50% NPK (5.50 ± 0.14) or 50% compost (5.00 ± 0.10). These treatments also significantly enhanced total fruit production per plant, with values of 181.14 ± 0.96 g and 176.59 ± 0.96 g, respectively. These correspond to yields of 94.19 t/ha and 91.52 t/ha, respectively, that are close to the yield achieved with the full NPK dose (C^+^_NPK_), which was 97.45 t/ha. Meanwhile, the highest average fruit weight (73.35 ± 1.80 g) was observed in plants treated with the full dose of NPK fertilizer (C^+^_NPK_).

The fruit shape coefficient (SC) values ranged from 0.74 for the negative control to 0.81 for the cyanobacterial consortium, individually (AN + NO) or amended with 50% NPK. According to the formula of Dossou et al. [33]. Fruit samples from treatments with individual strains (AN and NO) were classified as slightly flattened tomatoes (SC < 0.80), whereas those from treatments with consortium combined with 50% NPK or 50% Comp were classified as rounded tomatoes (SC > 0.80).

### 2.6. Effect of Cyanobacterial Bioinoculants on Tomato Biochemical Composition

**Figure 4:**
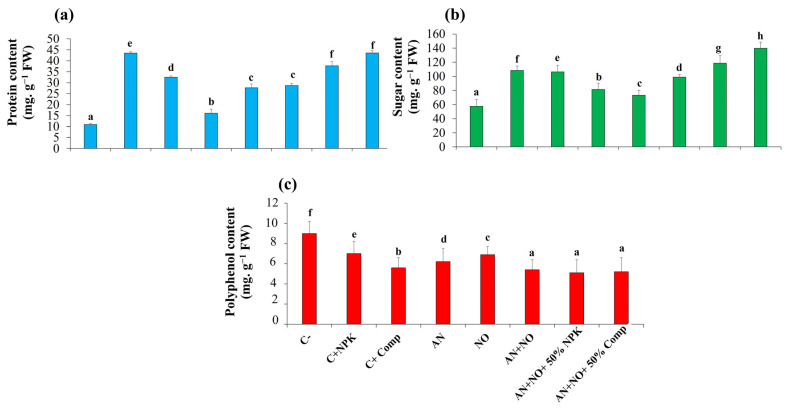
Influence of cyanobacterial treatments on biochemical constituents of tomato plants. Protein contents (**a**); sugar contents (**b**); polyphenol contents (**c**). FW: fresh weight; C^−^: negative control; C^−^: negative control; C^+^_NPK_: positive control, full dose of NPK (134 kg N/ha, 56 kg P/ha, and 276 kg K/ha); C^+^_Comp_: positive control, full dose of organic compost (250 g/kg); AN: fresh biomass of *Anabaena cylindrica*; NO: fresh biomass of *Nostoc punctiforme*; AN + NO: consortium of *Anabaena cylindrica* + *Nostoc punctiforme*; AN + NO + 50% NPK: consortium of *Anabaena cylindrica* + *Nostoc punctiforme* + 50% NPK; AN + NO + 50% Comp: consortium of *Anabaena cylindrica* + *Nostoc punctiforme* + 50% compost. Data are presented as mean ± SE (*n* = 3); means with different lowercase letters are statistically different according to Tukey’s HSD test (*p* < 0.05).

The results presented in Figure 4 demonstrate that all treatments significantly enhanced sugar and protein concentrations in tomato shoots compared to the negative control (C^−^) (*p* < 0.05). The most substantial enhancement was found in plants inoculated with consortium AN + NO + 50% Comp, which showed an increase of 299.17% in protein content and 142.72% in sugar content. In contrast, polyphenol concentrations were significantly lower in plants inoculated with cyanobacteria compared to the uninoculated plants (C^−^) (*p* < 0.05). The highest polyphenol content was recorded in the non-inoculated plants (9.04 ± 0.05 mg gallic acid equivalent g^−1^ FW), while the lowest contents (5.09 ± 0.09 and 5.15 ± 0.06 mg gallic acid equivalent g^−1^ FW) were observed in plants inoculated with the cyanobacterial consortium combined with 50% NPK or 50% organic compost, respectively.

### 2.7. Effect of Cyanobacterial Bioinoculants on Tomato Physiological Properties

**Figure 5:**
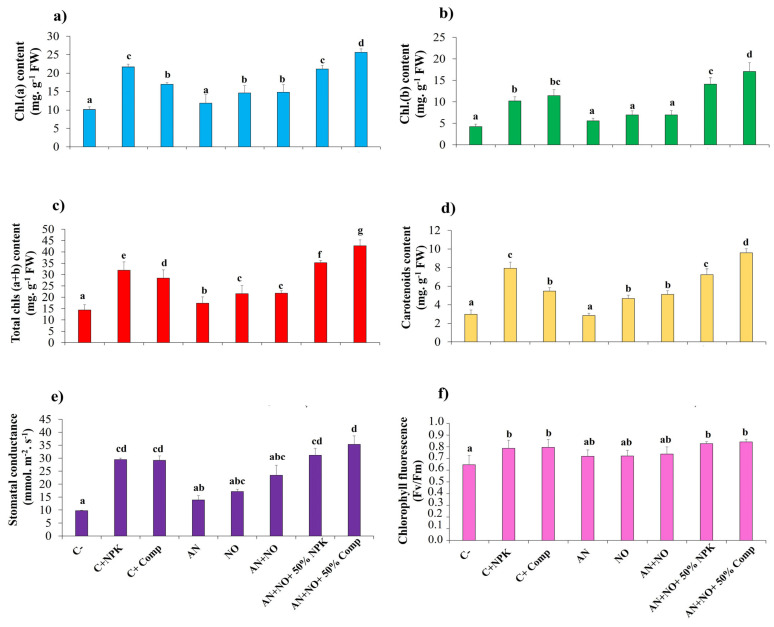
Influence of cyanobacterial treatments on tomato physiological traits. Chl.(*a*): chlorophyll *a* (**a**); Chl.(*b*): chlorophyll *b* (**b**); Total *chls* (*a + b*): total chlorophyll (**c**) and carotenoids (**d**); stomatal conductance (mmol·m^−2^·s^−1^) (**e**); chlorophyll fluorescence (Fv/Fm) (**f**). FW: fresh weight; C^−^: negative control; C^+^_NPK_: positive control, full dose of NPK (134 kg N/ha, 56 kg P/ha, and 276 kg K/ha); C^+^_Comp_: positive control, full dose of organic compost (250 g/kg); AN: fresh biomass of *Anabaena cylindrica*; NO: fresh biomass of *Nostoc punctiforme*; AN + NO: consortium of *Anabaena cylindrica* + *Nostoc punctiforme*; AN + NO + 50% NPK: consortium of *Anabaena cylindrica* + *Nostoc punctiforme* + 50% NPK; AN + NO + 50% Comp: consortium of *Anabaena cylindrica* + *Nostoc punctiforme* + 50% compost. Data are presented as mean ± SE (*n* = 3); means with different lowercase letters are statistically different according to Tukey’s HSD test (*p* < 0.05).

The results showed that the application of cyanobacterial strains, either alone or in combination, improved the physiological traits of tomato plants compared to non-inoculated plants (Figure 5). Plants inoculated with the AN + NO consortium combined with 50% NPK or 50% compost exhibited the highest content for chlorophyll *a* (21.15 ± 0.07 and 25.64 ± 0.47 mg·g^−1^ FW), chlorophyll *b* (14.11 ± 0.23 and 17.07 ± 0.26 mg·g^−1^ FW), total chlorophyll (35.26 ± 0.16 and 42.71 ± 0.21 mg·g^−1^ FW), and carotenoids (7.23 ± 0.15 and 9.58 ± 0.28 mg·g^−1^ FW), respectively (Figure 5a–d). These treatments also resulted in the highest increases in stomatal conductance (31.13 ± 2.67 and 35.31 ± 3.33 mmol·m^−2^·s^−1^) and chlorophyll fluorescence (0.83 ± 0.02 and 0.84 ± 0.02 Fv/Fm) compared to the other treatments. Interestingly, for both cyanobacterial combined treatments, all physiological parameters were significantly higher (*p* < 0.05) than those of the positive controls containing full doses of NPK (C^+^_NPK_) or organic compost (C^+^_Comp_), except for chlorophyll *a* and carotenoids (Figure 5e,f).

### 2.8. Effect of Cyanobacterial Bioinoculants on Tomato Mineral Properties

**Figure 6:**
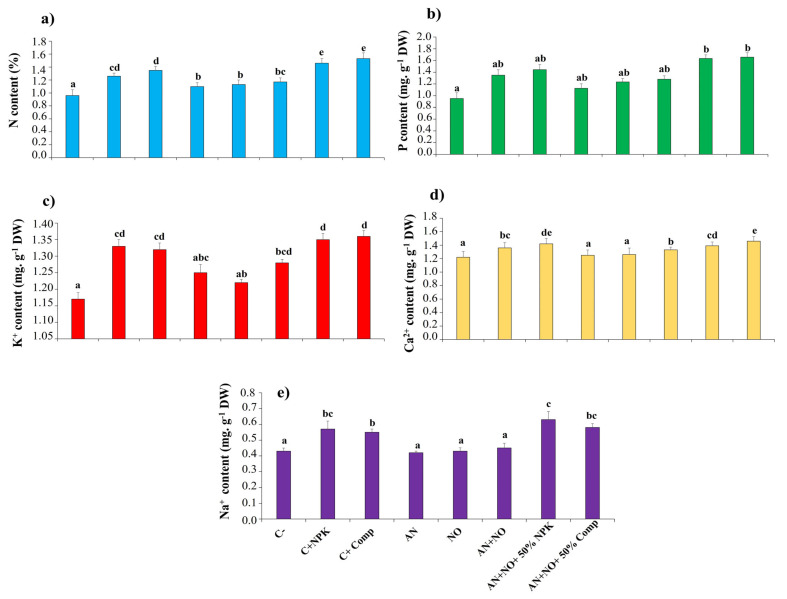
Influence of cyanobacterial treatments on mineral content of tomato plant shoots. N: nitrogen (**a**); P: phosphorus (**b**); K^+^: potassium (**c**); Ca^2+^: calcium (**d**); and Na^+^: sodium (**e**). DW: dry weight; C^−^: negative control; C^+^_NPK_: positive control, full dose of NPK (134 kg N/ha, 56 kg P/ha, and 276 kg K/ha); C^+^_Comp_: positive control, full dose of organic compost (250 g/kg); AN: fresh biomass of *Anabaena cylindrica*; NO: fresh biomass of *Nostoc punctiforme*; AN + NO: consortium of *Anabaena cylindrica* + *Nostoc punctiforme*; AN + NO + 50% NPK: consortium of *Anabaena cylindrica* + *Nostoc punctiforme* + 50% NPK; AN + NO + 50% Comp: consortium of *Anabaena cylindrica* + *Nostoc punctiforme* + 50% compost. Data are presented as mean ± SE (*n* = 3); means with different lowercase letters are statistically different according to Tukey’s HSD test (*p* < 0.05).

The mineral status of tomato plants improved following inoculation with fresh biomass of cyanobacterial strains, either alone or in combination (Figure 6). Treatments with the combined consortia (AN + NO + 50% NPK and AN + NO + 50% compost) proved to be the most effective bioinoculants, sometimes exceeding the values of the positive controls. Specifically, the nitrogen content in the shoots exceeded 1.40 mg kg^−1^ DW for the first treatment (AN + NO + 50% NPK) and 1.50 mg kg^−1^ DW for the second treatment (AN + NO + 50% compost). A similar trend was observed for phosphorus content, which exceeded 1.60 mg·kg^−1^ DW, as well as for Na^+^ (above 0.50 mg kg^−1^ DW), K^+^ and Ca^2+^ above 1.30 mg kg^−1^ DW in plants subjected to both treatments (Figure 6).

### 2.9. Effect of Cyanobacterial Bioinoculants on Tomato Fruit Quality

#### 2.9.1. Effect on Fruit Biochemical Parameters

All cyanobacteria treatments resulted in an improvement in the biochemical parameters of tomato fruits, including protein, sugar, polyphenol, and vitamin C content, compared to the negative control (Table 4). The greatest improvement was noted in plants inoculated with the *A. cylindrica* and *N. punctiforme* consortium combined with 50% compost (AN + NO + 50% Comp), which recorded protein content of 19.26 ± 0.05 mg g^−1^ FW, total sugars of 50.57 ± 2.00 mg g^−1^ FW, polyphenols of 18.32 ± 1.22 mg g^−1^ DW, and vitamin C of 30.40 ± 0.36 mg 100 g^−1^ FW.

#### 2.9.2. Effect of Cyanobacterial Bioinoculants on Physicochemical Parameters of Tomato Fruits

The analysis of physicochemical parameters of tomato fruits indicated that cyanobacterial bioinoculants led to a significant decrease (*p* < 0.05) in total titratable acidity and a significant increase (*p* < 0.05) in dry matter content. Additionally, there was a slight increase in pH values, though this change was not statistically significant (*p* > 0.05) compared to the negative control (Table 5). Furthermore, these treatments positively influenced the mineral content of the fruits, particularly phosphorus (P), calcium (Ca^2+^), potassium (K^+^), and sodium (Na^+^). The highest contents of P (3.03 ± 0.00 and 3.32 ± 0.00 mg g^−1^), K^+^ (4.60 ± 0.04 and 4.75 ± 0.03 mg·g^−1^), Na^+^ (0.63 ± 0.01 and 0.62 ± 0.00 mg g^−1^), and Ca^2+^ (3.06 ± 0.00 and 2.92 ± 0.04 mg g^−1^) were recorded in plants inoculated with the cyanobacterial consortium in the presence of 50% compost or 50% NPK, respectively.

### 2.10. Correlation Analysis of Growth, Biochemical, and Physiological Traits in Tomato Shoots and Fruit Quality

**Figure 7:**
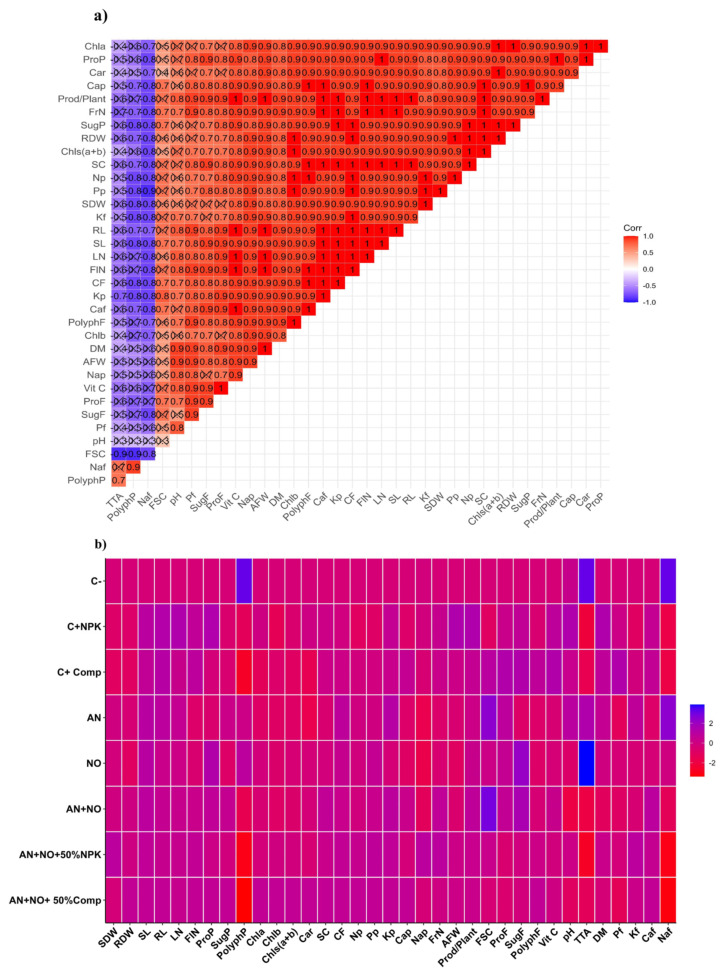
Pearson correlation matrix of physiological, biochemical, and biometric traits in tomato plants, as well as yield parameters and biochemical attributes of tomato fruits (**a**), and heatmap analysis of shoot and fruit traits in tomato plants subjected to different treatments (**b**). C^−^: negative control; C^+^_NPK_: positive control, full dose of NPK (134 kg N/ha, 56 kg P/ha, and 276 kg K/ha); C^+^_Comp_: positive control, full dose of organic compost (250 g/kg); AN: fresh biomass of *Anabaena cylindrica*; NO: fresh biomass of *Nostoc punctiforme*; AN + NO: consortium of *Anabaena cylindrica* + *Nostoc punctiforme*; AN + NO + 50% NPK: consortium of *Anabaena cylindrica* + *Nostoc punctiforme* + 50% NPK; AN + NO + 50% Comp: consortium of *Anabaena cylindrica* + *Nostoc punctiforme* + 50% compost. The growth, nutrition, fruit yield, and quality variables are represented in red. The eight treatments are given in blue. RDW, root dry weight; SDW, shoot dry weight; SL, shoot length; LN, leaves number; RL, root length; FlN, flower numbers/plant; FrN, fruit numbers/plant; AFW, average fruit weight; Prod/Plant, production/plant; FL, fruit lengths; FSC, fruit shape coefficient; SugP, sugar in the shoots; ProP, protein in the shoots; PolyphP, polyphenol in the shoots; SugF, sugar in the fruits; ProF, protein in the fruits; PolyphF, polyphenol in the fruits; Vit C, vitamin C; Car, carotenoids; Chls(a + b): total chlorophyll (a + b); Chl a, chlorophyll a; Chl b, chlorophyll b; SC, stomatal conductance; CF, chlorophyll fluorescence; Pp, phosphorus in the shoots; Np, nitrogen in the shoots; Cap, calcium in the shoots; Nap, sodium in the shoots; Kp, potassium in the shoots; Pf, phosphorus in the fruits; Caf, calcium in the fruits; Naf, sodium in the fruits; Kf, potassium in the fruits; TTA; total titratable acidity; DM; dry matter. In the heat map, the color gradient sets light purple as the minimum value (indicating negative correlation), dark red as the maximum value (indicating positive correlation), and white as the mid-range value, with a gradual transition (or gradient) between these endpoints. Note: ‘×’ indicates correlation is non-significant (*p* > 0.05).

The triangular correlation matrix (Figure 7a) illustrates the strength and direction of Pearson correlations among physiological, biochemical, and biometric traits in tomato plants, as well as yield parameters, biochemical attributes, and physicochemical properties of tomato fruits. The matrix reveals predominantly strong positive correlations (correlation coefficients close to 1.0, shown in red) between variables such as chlorophyll pigments (Chla, Chlb, and total Chl), productivity parameters (Prod/Plant and FrN), and various plant biochemical and mineral elements (Car, Cap, and ProP). These correlations suggest that improvements in one trait (e.g., photosynthetic pigment) are closely associated with enhancements in others (e.g., yield-related traits), indicating a coordinated physiological response among the measured traits. Only a few weak or negative correlations (indicated in blue to light purple) were observed, reflecting a generally consistent and synergistic pattern of trait behavior under the experimental conditions.

The heatmap (Figure 7b) displays the standardized responses of biochemical, physiological, and agronomic traits across the different treatment groups. Each row represents a treatment, and each column corresponds to a specific variable. The color gradient ranges from red (lower values) to blue (higher values), enabling clear visualization of the treatment effects on plant trait expression. Notably, the treatments C^+^_Comp_ (full dose of organic compost), C^+^_NPK_ (full dose of NPK), and AN + NO + 50% Comp (cyanobacterial consortium combined with 50% compost) exhibited elevated values (blue hues) in critical parameters such as chlorophyll content (Chla and Chls(a + b)), fruit productivity (Prod/Plant), and fruit antioxidants (PolyphF and Vit C). In contrast, the C^−^ (untreated control), as well as the individual applications of *N. punctiforme* (AN) and *A. cylindrica* (NO), showed reduced values (red to yellow) across most parameters. These findings suggest that combining compost or NPK with the cyanobacterial consortium, particularly in the AN + NO + 50% Comp treatment, was found to be positively correlated with important enhancements in plant growth and fruit quality compared to individual or untreated controls.

## 3. Discussion

Recent agricultural research has increasingly explored the potential of cyanobacteria-based biofertilizers as a sustainable and eco-friendly alternative to synthetic fertilizers and intensive farming practices [3,4,10]. These microorganisms demonstrate significant potential for enhancing crop growth, boosting yields, increasing resilience to abiotic stresses, and promoting sustainable agricultural production [7,13,34,35]. In line with this trend, the present study aims to examine the impact of a soil cyanobacteria-based bioinoculant consortium on soil fertility, tomato plant growth, biochemical and physiological traits, and fruit quality.

The characterization of the two indigenous soil cyanobacteria *A. cylindrica* and *N. punctiforme* revealed several promising physiological and biochemical traits, underscoring their potential as effective bioinoculants for enhancing soil fertility, growth, and yield in tomato crops. These strains demonstrated a strong capacity for nitrogen fixation, phytohormone production (such as auxins), exopolysaccharide secretion, protein synthesis, and phosphate solubilization. Additionally, these terrestrial cyanobacteria offer significant advantages, not only due to their ability to release bioactive compounds but also because of their minimal toxic risk. Importantly, no cyanotoxins, such as microcystins, which can pose serious hazards to the environment, human health, and soil microbiota, were detected.

The results demonstrated a significant improvement in soil physicochemical properties following the application of cyanobacterial bioinoculants. The cyanobacterial treatments led to a notable increase in organic carbon and macronutrient contents, including N, P, K^+^, Na^+^, and Ca^2+^. The most substantial enhancements were observed in soils treated with a consortium of *A. cylindrica* and *N. punctiforme* combined with either 50% compost or 50% NPK fertilizers. These modifications in the plant rhizosphere provide valuable insights into the ability of cyanobacteria to mitigate nutrient deficiencies and enhance crop growth. The decline in soil pH may be attributed to the secretion of extracellular substances, such as polysaccharides, peptides, and organic acids, which contribute to soil neutralization [36]. Furthermore, the production of organic acids during compost decomposition may further contribute to the reduction in soil pH, while the associated increase in salt concentration explains the observed rise in soil conductivity. These findings are consistent with research by Boutasknit et al. [37], who demonstrated that compost application results in a decrease in soil pH along with an increase in conductivity.

In addition, inoculation with fresh biomass of *A. cylindrica* and *N. punctiforme* enhances soil nutrient bioavailability, either directly or indirectly [38]. The observed increases in P, N, Ca^2+^, K^+^, and Na^+^ contents in the treated soils can be attributed to the decomposition of cyanobacterial biomass and the direct supplementation of these elements through compost and NPK amendment [3,5,37]. Furthermore, the application of *A. cylindrica* and *N. punctiforme* improves soil fertility and quality through various mechanisms, including nitrogen fixation, phosphate solubilization, and the production of extracellular polymeric substances (EPSs) [4,19,23,39]. These findings are consistent with numerous studies demonstrating that cyanobacterial inoculation enhances soil biological activity. This enhancement, in turn, improves the nutrient status of the rhizosphere by facilitating the mobilization and bioavailability of essential macro- and micronutrients, thereby promoting their uptake by plants [4,20,21,22,28].

The positive impact of cyanobacterial bioinoculants on soil physicochemical properties contributed to the improvement in plant traits, resulting in increased tomato growth and yield. The results indicated that the application of cyanobacterial bioinoculants, either individually or in consortium alone or with NPK or compost, improved the growth of tomato compared to the negative control. Statistical analysis revealed notable differences in tomato growth parameters after inoculation of the consortium, showing superior effects when combined with either 50% NPK or 50% compost. In some cases, these effects surpassed those of the positive controls, which received full doses of NPK (C^+^_NPK_) or organic compost (C^+^_Comp_). Biofertilizing with cyanobacteria has the potential to significantly reduce the need for chemical fertilizers and organic compost by up to 50%, while maintaining tomato plant growth. These results are in line with previous studies supporting the beneficial impacts of cyanobacterial treatments on plant growth [13,17,26,40]. The enhanced tomato growth observed in cyanobacteria-inoculated soils is often attributed to the richness of cyanobacterial biomass in macro- and microelements. The availability of these elements is an important factor in promoting plant growth. N, P, and K are the main essential macronutrients, which play a crucial role in crop nutrition [13,23].

Additionally, this growth can be linked to the production of certain growth regulators, such as auxins, by the two cyanobacterial species studied [7,12,26,35]. Hormonal analysis revealed differences in indole-3-acetic acid (IAA) levels between *A. cylindrica* and *N. punctiforme*. The *A. cylindrica* strain exhibited higher IAA levels (12.74 μg/mL) compared to *N. punctiforme* (4.16 μg/mL). Similar results were obtained by Gheda and Ahmed [26], who studied the hormonal effects of the cyanobacterial strains *Nostoc kihlmani* and *A. cylindrica* on wheat growth, including root length, shoot length, and dry weight enhancement. Auxin activity is known to induce or stimulate root cell elongation, cell division, xylem and phloem differentiation, adventitious root formation, and activation of certain enzymes [41,42].

This study also assessed the effect of different cyanobacterial treatments on tomato yield. The results showed a significant improvement in the yield of treated tomatoes compared to the untreated plants. The most effective treatment was soil inoculation with the consortium AN + NO + 50% Comp. These results are in line with studies by Bona et al. [43], who reported that the application of microalgae-based biofertilizers increased tomato yield by enhancing nutrient supply and bioavailability. The incorporation of organic and biological agents improves both the direct and indirect supply of essential elements, such as nitrogen (N) and phosphorus (P), which accelerate growth, reproduction, and protein synthesis, ultimately increasing crop yields [3,5,37,38,43]. Soil inoculation with the fresh biomass of two cyanobacterial strains, combined with the addition of either half the recommended dose of chemical or organic fertilizer, appears to be more efficient and, above all, more cost-effective, potentially reducing fertilizer use by 50%. In this regard, Pereira et al. [44] found that biofertilization with a consortium of nitrogen-fixing cyanobacteria (*Nostoc commune*, *Nostoc* sp., *Nostoc linckia*, and *Anabaena* sp.) reduced nitrogen fertilizer requirements by 50% while maintaining the same grain yield and rice quality as the full dose of chemical fertilizer. Similarly, Alvarez et al. [4] found that bioinoculation with *A. cylindrica* and a combination containing the green alga *Chlamydomonas* sp. supplied nitrogen to wheat plants, promoting their growth and yield. Their study demonstrated that these treatments could replace between 75% and 100% of the nitrogen typically supplied through urea without compromising grain yield.

A comparative analysis of greenhouse pot experiments revealed that cyanobacterial treatments led to significant differences in the biochemical, physiological, and mineral composition of treated plants compared to untreated plants. The results indicated an increase in total sugars and total proteins, along with a decrease in total phenols in treated plants. These observations are in agreement with those published in previous studies, which observed that cyanobacterial inoculation significantly enhanced biometric parameters and biochemical composition, including total sugars and total proteins [7,15,42,45]. The rise in protein content can be attributed to the ability of cyanobacterial strains to secrete auxin, which stimulates cell division, leading to greater plant biomass and increased protein synthesis activity [15,46]. Additionally, the increase in carbohydrate content may result from enhanced CO_2_ fixation [47].

Polyphenols are chemical compounds synthesized by plants as part of their defense mechanism against oxidative stress. They play a critical role in neutralizing reactive oxygen species (ROS) produced under various stress conditions [48]. Cyanobacterial bioinoculants, along with other soil microorganisms like mycorrhizal fungi and plant growth-promoting rhizobacteria (PGPR), have been shown to mitigate both biotic and abiotic stresses [18,34]. In this research, the evaluation of stress-related parameters indicated that the application of cyanobacterial bioinoculants did not induce oxidative stress in tomato plants. This conclusion was supported by measurements of antioxidant capacity, specifically through the quantification of polyphenol content. The results showed a significant reduction in polyphenol levels in treated tomato plants compared to the untreated control. These findings are consistent with recent studies by Roque et al. [32], who reported that a mixture of *Trichocoleus* sp., *Nodosilinea* sp., *Microcoleus* sp., and *Klebsormidium* sp. exhibited biostimulant potential for *Arabidopsis thaliana* and *Lolium multiflorum*. Their study also confirmed that stress-related parameters indicated that the consortium did not trigger a stress response in plants.

Improving the biochemical properties of tomato plants can enhance photosynthetic efficiency. In this study, soil inoculation with a consortium of both cyanobacterial strains, combined with the addition of 50% compost or NPK, significantly improved key photosynthetic parameters in tomato plants. These included chlorophyll fluorescence (Fv/Fm), stomatal conductance, and the concentrations of carotenoids, Chl *b*, and Chl *a*, indicating a well-functioning photosynthetic system and efficient CO_2_ assimilation [49]. Enhanced photosynthesis promotes the synthesis of proteins and total sugars, which positively influence tomato plant growth and yield. This improvement may also be attributed to a protective mechanism that reduces degradation of chlorophyll [50]. Furthermore, cyanobacterial biomass contains a unique set of bioactive compounds, notably plant growth regulators, which can increase leaf chlorophyll content, delay senescence, and reduce transpiration [51].

Regarding plant mineral status, the findings revealed a significant enhancement in the mineral content (P, N, K, Ca, and Na) of tomato plants treated with cyanobacterial bio-inoculants compared to uninoculated plants. The highest concentrations of these nutrients were recorded in plants inoculated with the cyanobacterial consortium + 50% compost. These outcomes are consistent with those reported by Gheda and Ahmed [26], who demonstrated that inoculation with fresh biomass of *Anabaena cylindrica* and *Nostoc kihlmani* enhanced growth and increased NPK content in wheat plants. The synergistic effect between *A. cylindrical* and *N. punctiforme* may be explained by the compost’s dual role in providing essential nutrients to tomato plants and fostering favorable conditions for the proliferation of cyanobacteria and other beneficial soil microbes [5]. Cyanobacteria are known to improve plant mineral nutrition effectively [52]. The higher nitrogen content observed in plants treated with cyanobacteria may result from the increased fixation of atmospheric nitrogen by intact cyanobacterial cells, which can fix up to 25 kg N/ha [53], subsequently transferring this nitrogen to tomato tissues. This increase may also be linked to the presence of macro-elements inherently contained within the cells of the used cyanobacterial strains. In addition to their nitrogen-fixing and phosphorus-solubilizing abilities, both strains are capable of secreting auxin, which promotes root growth. By initiating adventitious and lateral roots and stimulating their cell division and elongation, auxin enhances plant nutrient uptake [26].

The results of this study demonstrated that the application of cyanobacterial treatments significantly enhances the quality and nutritional value of tomato fruits. The most effective treatments involved plant inoculation with the amended cyanobacterial consortium. Soil inoculation with these treatments led to a significant increase in total sugar and protein content. These findings align with those of Gashash et al. [54], who reported that inoculation with cyanobacteria and plant growth-promoting rhizobacteria (*Bacillus amyloliquefaciens* and *Bacillus subtilis*) significantly improved the growth, yield, and fruit quality of tomatoes. The increase in fruit protein content was linked to the rise in N content [45], while the rise in sugar content can be explained by the action of cyanobacteria bioregulators, which stimulate the photosynthetic activity of tomato plants [46]. Moreover, antioxidant activities, including total phenol and ascorbic acid levels, were positively influenced by these biofertilizing treatments. The synthesis and accumulation of antioxidant compounds, such as ascorbic acid, may result from the direct or indirect effects of biofertilizer applications on the biosynthesis and renewal of antioxidants in plant tissues [55].

Additionally, the findings of this study demonstrated a significant improvement in the physicochemical parameters of tomato fruits following the application of cyanobacterial treatments. These enhancements included a reduction in total titratable acidity, an increase in pH values, higher dry matter content, and elevated mineral element contents compared to the non-inoculated control. These findings are supported by a study suggesting that the use of biofertilizers helps plants absorb sufficient water from the soil, resulting in a decrease in total titratable acidity in the fruit [56]. The increase in mineral element contents in tomato fruits can be explained by several factors, particularly the presence of bioactive compounds in cyanobacterial biomass, such as proteins, carbohydrates, and free amino acids, which may enhance sink strength and influence the movement of nutrient substrates, including minerals, within the plant [57]; improved mineral uptake due to the stimulation of root growth [58]; and increased expression of nutrient transporters in cells [58]. Furthermore, the incorporation of compost as an organic fertilizer plays a critical role in enhancing fruit quality. This is due to its ability to improve both the quantity and quality of soil nutrient supply. Compost application not only stimulates plant growth, physiology, and yield but also positively impacts fruit quality parameters. These outcomes are consistent with previous studies highlighting the multifaceted benefits of compost in sustainable agricultural practices [59,60].

**Figure 8:**
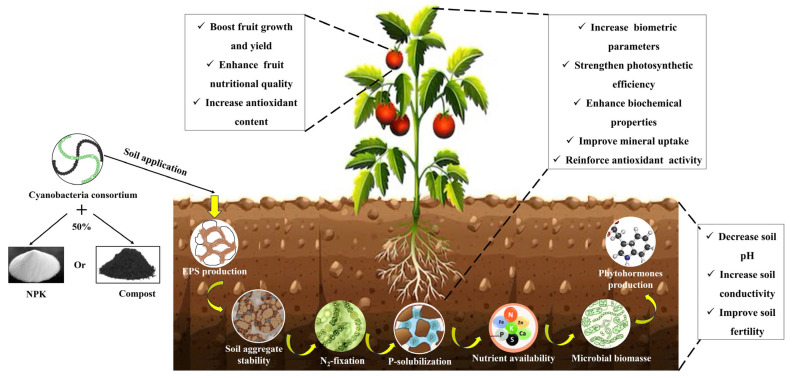
Suggested mechanism of the regulatory network involved in tomato growth in response to cyanobacteria consortium, compost, and NPK treatments. Dashed lines represent the findings of this study. Solid yellow lines indicate mechanisms reported in the literature (Figure created with Biorender.com).

The results highlight a physiological and biochemical switching mechanism triggered by the cyanobacterial consortium and provide further support for the hypothesis illustrated in Figure 8. This mechanism suggests that the cyanobacterial consortium combined with 50% NPK or compost acts at multiple levels, including the soil, plant, and fruit. This study showed significant improvements in all measured parameters in tomatoes grown in soils treated with cyanobacterial biofertilizers, particularly when using the consortium in combination with 50% compost or NPK. These enhancements in soil fertility, plant growth, mineral uptake, physiological and biochemical traits, and fruit yield and quality, along with a reduction in polyphenol content, are likely due to the synergistic effects between cyanobacteria and conventional fertilizers. Specifically, cyanobacteria may enhance soil fertility by modulating microbial biomass, fixing atmospheric nitrogen, and solubilizing phosphate, thus increasing the availability of nitrogen and phosphorus for plant uptake. Additionally, the production of EPS may contribute to improved soil structure. Cyanobacteria can also stimulate plant growth and yield through phytohormone secretion and the presence of bioactive compounds (e.g., proteins, carbohydrates…), which may enhance fruit nutritional quality. Compost contributes essential macronutrients (N, P, and K) while also improving soil health through increased organic matter content and enhanced soil structure. NPK fertilizers promote vigorous vegetative growth by supplying nitrogen, enhance fruit formation via potassium, and contribute to higher overall yield. The overall trend observed in our data suggests that soil amendments, especially those involving the cyanobacterial consortium, consistently improve plant development, soil properties, and fruit yield and quality.

## 4. Materials and Methods

### 4.1. Cyanobacterial Strains and Culture Conditions

Two indigenous cyanobacteria strains, *Anabaena cylindrica* (MACC-CY00070) and *Nostoc punctiforme* (MACC-CY00069), were isolated from enriched soil cultures using serial dilution and repeated streak platting, as described in our previous study [61]. Briefly, soil samples were serially diluted (from 10^−1^ to 10^−5^) and inoculated onto both solid and liquid Z8 medium [62]. This medium consisted of (per liter) NaNO_3_ (46.7 g), Ca(NO_3_)_2_·4H_2_O (5.9 g), MgSO_4_·7H_2_O (2.5 g), K_2_HPO_4_ (3.1 g), Na_2_CO_3_ (2.1 g), FeCl_3_ (2.8 g), and Na_2_EDTA (3.9 g). It also included a trace metal solution comprising essential micronutrients: Na_2_WO_4_·2H_2_O (3.3 mg), (NH_4_)_6_Mo_7_O_24_·4H_2_O (8.8 mg), KBr (12 mg), KI (8.3 mg), ZnSO_4_·7H_2_O (28.7 mg), Cd(NO_3_)_2_·4H_2_O (15.5 mg), Co(NO_3_)_2_·6H_2_O (14.6 mg), CuSO_4_·5H_2_O (12.5 mg), NiSO_4_·(NH_4_)_2_SO_4_·6H_2_O (19.8 mg), Cr(NO_3_)_3_·9H_2_O (4.1 mg), KAl(SO_4_)_2_·12H_2_O (47.4 mg), V_2_O_5_ (0.89 mg), H_3_BO_3_ (3.1 g), and MnSO_4_·H_2_O (160 mg). Cultures were maintained under controlled conditions at 25 ± 2 °C, with a photon flux density of 62 μmol·m^−2^·s^−1^, a 15 h/9 h light/dark cycle, and continuous aeration provided by an air pump supplying ambient (non-enriched) air for 8–12 days. The monoculture strains were obtained through successive transfers onto fresh medium. The *A. cylindrica* strain was isolated from non-agricultural soil collected at Oukaimeden, located in the High Atlas Mountains of Marrakesh, Morocco (N 31°11.620′ W 007°51.207′), while *N. punctiforme* was isolated from forest soil near the city of Essaouira (N 31°32′11.3″ W 009°28′44.4″). The two cyanobacterial strains were taxonomically identified based on their morphological characteristics observed under a light microscope (Motic BA210, Xiamen, China), in accordance with the criteria described by Komárek [63], and through molecular identification using 16S rRNA gene sequencing. The monocyanobacterial cultures of *A. cylindrica* and *N. punctiforme* were cultivated in a closed batch system using 500 mL Erlenmeyer flasks containing 450 mL of sterile Z8 medium without N to induce nitrogen fixation [62]. Cultures were grown under the same controlled conditions described above. Stock cultures of both strains were kept in exponential growth through periodic subculturing, and their purity was determined by microscopy. Subsequently, the growth of each strain was performed in triplicate cultures using 250 mL Erlenmeyer flasks, each containing 200 mL of Z8 medium, and inoculated with fresh cyanobacterial inoculum to reach a low initial OD_750_ of 0.05, as measured by a UV-Visible spectrophotometer (Varian, Cary, 50 Scan, Palo Alto, CA, USA). Cultures were incubated for 20 days under the aforementioned conditions. Growth was monitored daily by cell counting at 400× magnification using a light microscope, following Mischke et al. [64]. The growth rate (μ) was determined using the classical Monod equation [65]:

µ (day^−1^) = [ln(N_2_) − ln(N_1_)]/Δt

where Δt corresponds to the time interval (t_2_ − t_1_) and N_1_ and N_2_ represent biomass concentrations at times t_2_ and t_1_, respectively.

### 4.2. Physiological Traits of Cyanobacterial Strains

Some physiological characteristics of *N. punctiforme* and *A. cylindrica* strains were assessed, including their ability to produce exopolysaccharides (EPSs) and indole-3-acetic acid (IAA), to fix atmospheric nitrogen (N_2_), and to solubilize phosphate. For EPS quantification, the extraction was performed using a dual ultrasonic and heat method, as detailed by Strieth et al. [66], and the EPS content was quantified gravimetrically. IAA production ability of each strain was measured through a colorimetric Salkowski assay. Following a 15-day incubation, 2 mL of Salkowski reagent was mixed with 1 mL of the *N. punctiforme* and *A. cylindrica* culture supernatant (grown in Z8 medium supplemented with 100 mg/L L-tryptophan). The resulting solution was then analyzed spectrophotometrically to quantify IAA contents [67]. Nitrogen fixation capacity was measured using a qualitative assay with Z8 liquid medium (without nitrogen). Strain growth was monitored to check their ability to fix atmospheric nitrogen (N_2_) [68]. The phosphate solubilization capacity was evaluated using a qualitative assay. The strains were streaked onto Z8 agar Petri dishes supplemented with 0.3% (*w*/*v*) tricalcium phosphate as the insoluble phosphate source. The Petri dishes were then incubated at 26 ± 2 °C for 15 days. Phosphate solubilization was indicated by the appearance of clear halos surrounding the cyanobacterial colonies, reflecting the dissolution of tricalcium phosphate into a soluble form [68].

### 4.3. Cyanobacterial Biomass Production and Characterization

Biomass production was carried out in a closed batch system using the methodology described by Hakkoum et al. [7]. For biomass characterization, 5 g of fresh cyanobacterial biomass was suspended and mixed in 25 mL of distilled water. The electrical conductivity (EC) and pH of the suspension were determined using a conductivity meter (Cond 1970i WTW GmbH, Weilheim, Germany) and a pH meter (pH 1970i WTW GmbH, Weilheim, Germany), respectively. To analyze the biochemical and mineral compounds, the biomass fraction of *A. cylindrica* and *N. punctiforme* was lyophilized using a freeze dryer (Christ^®^ Alpha 1-4 LSC basic, Osterode am Harz, Germany). Total soluble sugar content was determined via the phenol-sulfuric acid method, as described by Dubois et al. [69], using the phenol-sulfuric acid protocol. Concurrently, protein quantification was performed according to the Bradford colorimetric assay [70], with bovine serum albumin (BSA) serving as the calibration standard. Total polyphenols and flavonoids were analyzed using the colorimetric methods described by Singleton et al. [71] and Bahorun et al. [72], respectively. Total organic carbon (TOC) content was measured by kiln calcination at 650 °C using the method of Haug [73]. The mineral microelements Na^+^, Ca^2+^, and K^+^ were assessed according to the method of Pequerul et al. [74] using a flame spectrophotometer (Flame photometer, model AFP-100, Sedico Company, Nicosia, Cyprus). The total Kjeldahl nitrogen (TKN) and total phosphorus in the cyanobacterial biomass were quantified using the AFNOR standards described by Rodier et al. [75] and the protocol of Murphy and Riley [76], respectively. The content of phycobiliproteins (allophycocyanin, phycoerythrin, and phycocyanin) in cyanobacterial strains was quantified using a spectrophotometer during their exponential growth phase, following the method described by Bennett and Bogobad [77]. Briefly, 10 mL of the strain culture was sonicated and then centrifuged. The obtained pellet was carefully resuspended in 6 mL of a buffer solution (saline buffer). The phycobiliprotein content was estimated by measuring absorbance at wavelengths of 562 nm, 615 nm, and 652 nm. To evaluate the safety and toxicity of *A. cylindrica* and *N. punctiforme*, the production of cyanotoxins, specifically microcystins, was assessed using the phosphatase inhibition assay (PP1). The analysis was conducted at AgroParisTech, University Paris-Saclay, France, following the method described by Bouaïcha et al. [78].

### 4.4. Tomato Pot Experiments and Bioassay

#### 4.4.1. Soil Sampling and Physicochemical Analysis

The agricultural soil used in this experiment was collected from unplanted farmland in the Ait Ourir locality (Marrakesh region) (N 31°33′54″ W 7°40′05″), which had not been amended with conventional fertilizers. The soil has been air-dried and sieved (<2 mm) to remove gravel and debris before analysis. The texture is predominantly clay, consisting of 49% clay, 25.3% silt, and 25.7% sand. The soil properties analysis revealed a slightly alkaline pH, low conductivity, and low contents of organic carbon (TOC), total nitrogen (TKN), calcium (Ca^2+^), and micronutrient (Na^+^) (Table 6).

To assess the fertility status of the soil, the physicochemical properties were analyzed after cyanobacterial inoculation. Soil conductivity and pH were measured using a conductivity meter and pH meter, respectively. Total organic carbon (TOC) was determined using the methodology developed by Aubert [79]. Total Kjeldahl nitrogen (TKN) was assessed using the standard Kjeldahl digestion method [75]. Available phosphorus (P) was estimated through a colorimetric technique as documented by Olsen et al. [80]. Mineral elements potassium, calcium, and sodium were performed through flame photometry following the protocol established by Pequerul et al. [74]. Finally, the texture of the soil was determined according to the Robinson pipette method as outlined by Baize [81].

#### 4.4.2. Conventional Fertilizers

The organic fertilizer (compost) utilized in this study was prepared following the method detailed by Mobaligh et al. [82]. It was produced from by-products of argan oil extraction (pulp and oil cake). The composting process was conducted over three months at the Faculty of Sciences Semlalia, Marrakesh. The physicochemical properties of this compost were characterized, revealing the following characteristics: pH: 8.1, EC: 4.5 mS cm^−1^, humidity: 44.24%, TOC: 27.90%, TKN: 1.67%, C/N: 16.71, and P: 0.45 g kg^−1^. The treatment by the compost was applied to the soil at a rate of 2.5% (*w*/*w*), corresponding to half of the recommended dose as outlined by Mobaligh et al. [82]. The mineral fertilizer NPK was applied to the soil at half the recommended dose for tomato crops, which corresponds to 134 kg N/ha, 56 kg P/ha, and 276 kg K/ha, as specified by Elalaoui [83]. The composition of the NPK fertilizer was 12%N (as NH_4_NO_3_), 14%P (as P_2_O_5_), and 8%K (as K_2_O).

#### 4.4.3. Experimental Design

Prior to the experiment of inoculating the soil with cyanobacterial strains, a preliminary test was performed to check whether there was any antagonism between *A. cylindrica* and *N. punctiforme* strains. The antagonism test was determined by an agar anticyanobacterial assay. The two strains were co-cultured, in triplicate, on solid Z8 medium (1.5% agar) onto the same Petri dish at three cm apart and then incubated for 14 days in the culture chamber under the conditions mentioned above. The results showed no clear inhibition zone between the two strains, suggesting the absence of antagonism and possible compatibility between these two cyanobacteria.

Tomato seeds (*Solanum lycopersicum* Mill. cv. Campbell 33) were washed with distilled water and disinfected in a 4% sodium hypochlorite solution for 10 min with agitation. To remove any traces of sodium hypochlorite, the seeds were rinsed five times with sterile distilled water (each wash lasting at least 10 min) [84]. Subsequently, the seeds were germinated in Petri dishes and incubated at 28 °C in darkness for 5 days. After germination, the seedlings were transferred to plastic trays containing sterile peat. Following one month of growth, uniform and healthy tomato plants were transplanted into plastic pots, each containing 5 kg of agricultural soil that had been sterilized at 180 °C for 3 h. For the inoculated pots, the soil surface was mixed with different treatments prepared using fresh cyanobacterial biomass. Eight treatments were applied once, as outlined below:

1. C^−^, control, non-bioinoculated, and non-amended soil.
2. C^+^_min_, positive control, soil fertilized by a full dose of NPK (3.5 g NPK/kg soil).
3. C^+^_org_, positive control, soil fertilized by a full dose of compost (50 g/kg soil).
4. AN, 10 g of *A. cylindrica* (AN) (2 g fresh biomass/kg soil).
5. NO, 10 g of *N. punctiforme* (NO) (2 g fresh biomass/kg soil).
6. AN + NO, 10 g of consortium, 5 g of *A. cylindrica* + 5 g of *N. punctiforme* (2 g fresh biomass/kg soil).
7. AN + NO + 50% NPK, 10 g of consortium + 50% NPK (1.75 g NPK/kg soil).
8. AN + NO + 50% Compost, 10 g of consortium + 50% compost (25 g/kg soil).

The pots were placed in semi-controlled greenhouse conditions (daylight photon flux density ranging from 500 to 750 μmol·m^−2^·s^−1^, temperature varying between 21 °C and 30 °C, and a relative humidity of 60% to 70%). The treatments were arranged in a randomized complete block design with six replicates per treatment. All pots were watered regularly with tap water as needed. The experiment was carried out over a duration of five months.

### 4.5. Tomato Plant Growth and Yield Parameters Measurement

Several biometric parameters of tomato plants were evaluated after five months of cultivation, at the mature growth stage. For each treatment, the measurements were performed in six pot replicates, and in each pot, one plant was observed. The assessed parameters included shoot and root lengths, number of leaves and flowers, and fresh and dry weights of both shoots and roots. To determine the dry weight, the roots were carefully separated and washed to eliminate any adhering soil particles. The lengths of both roots and shoots (in cm) were manually measured. Subsequently, fresh weights (in g) of shoot and root samples were recorded prior to drying them for 72 h at 70 °C, until a constant weight was obtained, representing the dry weight (in g). Fruit productivity was evaluated by measuring the number of fruits per plant, the total fruit production per plant (total weight of fruits per plant in g), and the average fruit weight, as described by Alavoine et al. [85]. Additionally, the fruit-shape index (L/D) was calculated as the ratio of fruit length to diameter, following the method outlined by Dossou et al. [33].

### 4.6. Tomato Plant Biochemical and Mineral Analyses

Fresh leaf and shoot samples were used to evaluate biochemical parameters. Protein levels were quantified following the Bradford [70] method. Total soluble sugars were measured based on the procedure of Dubois et al. [69]. Total phenolic content was dosed using the Folin–Ciocalteu reagent, as reported by Singleton et al. [71]. Total flavonoid was quantified using the aluminum trichloride assay, with catechin as the standard [72]. The mineral composition of the plants was analyzed using dried shoots. Concentrations of potassium (K^+^), sodium (Na^+^), and calcium (Ca^2+^) were quantified in acid-digested samples via a flame photometer (Flame photometer, model AFP-100, Sedico Company, Nicosia, Cyprus) according to the methodology of Pequerul et al. [74]. Phosphorus level was measured following the colorimetric procedure of Murphy and Riley [76]. Additionally, total nitrogen was evaluated via the Kjeldahl digestion method, as detailed by Bremner [86].

### 4.7. Tomato Plant Physiological Analyses

Photosynthetic pigment concentrations, including carotenoids, total chlorophyll, chlorophyll *a*, and chlorophyll *b*, were quantified in fresh leaf samples. Extraction was performed using 95% acetone, followed by spectrophotometric analysis (UV-visible) according to the methodology detailed by Upadhyaya et al. [87]. Stomatal conductance (*g_s_*) was measured using a portable steady-state diffusion porometer (Leaf Porometer LP1989, Decagon Devices, Inc., Washington, DC, USA) on the abaxial side of the leaves. Six measurements were taken per treatment between 9:00 a.m. and 11:30 a.m. Chlorophyll fluorescence (F_v_/F_m_) was measured on the third youngest fully expanded sunlit leaves using a hand-held fluorometer (OPTI-SCIENCE, OS30p, Hudson, NY, USA). Prior to measurement, the leaves were dark-adapted for 30 min using specialized leaf clips. The maximum quantum efficiency of photosystem II (F_v_/F_m_) was calculated using the formula F_v_/F_m_ = (F_m_ − F_0_)/F_m_, where F_0_ is the minimal fluorescence, F_m_ is the maximal fluorescence of dark-acclimated leaves, and F_v_ is the variable fluorescence [88].

### 4.8. Tomato Fruit Quality Characteristics

The protein and soluble sugar content in the fruits were measured using the methods described by Bradford [70] and Dubois et al. [69], respectively. Total phenolic content, expressed as gallic acid equivalent, was evaluated following the protocol reported by Singleton et al. [71]. Total phosphorus was determined using the method described by Murphy and Riley [76]. Mineral contents (Na^+^, K^+^, and Ca^2+^) were analyzed using the flame photometric method [74]. Ascorbic acid was quantified using a titrimetric method [89]. Briefly, 0.5 g of fresh fruit was finely ground in a mortar and mixed with 3 mL of 2% hydrochloric acid. The vitamin extract was centrifuged, and 1 mL of the supernatant was combined with 3 mL of distilled water and 3 drops of 0.5% starch solution as an indicator. The mixture was then titrated with 0.01 N iodine solution. Total titratable acidity (TTA) was dosed in tomato juice following the method established by Alavoine et al. [85]. Briefly, 10 mL of juice was filtered and homogenized, and 4 drops of phenolphthalein were added as an indicator. The solution was titrated with 0.1 M sodium hydroxide (NaOH) dropwise until a pink color appeared. The results were expressed as a percentage of citric acid. The dry matter content of tomato fruit was determined using the oven-drying method according to Rouphael et al. [90]. A 10 g sample of tomato pulp from each treatment was finely chopped, weighed, and placed in a pre-weighed aluminum dish. The sample was dried in an oven at 70 °C for 6–7 h until a stable weight was obtained. The dry matter content was expressed as a percentage (%) of the initial fresh weight. The pH of tomato juice was determined using a pH meter.

### 4.9. Statistical Analysis

All analyses were performed in three replicates. The results are given as mean ± standard error (SE). Statistical analysis was conducted using SPSS software (version 22.0, IBM Corp., Armonk, NY, USA). Treatment effects on measured parameters were evaluated through one-way analysis of variance (ANOVA), with significant differences (*p* < 0.05) between means determined by Tukey’s honestly significant difference (HSD) post hoc test. Furthermore, a correlation analysis at the 0.05 significance level was conducted between measured plant traits using the Rstudio (version 4.4.2) [91] via the corrplot package (version 0.95) [92], and the graphical representation of the correlogram was performed using the ggcorrplot package (version 0.1.4.1) [93]. Moreover, a heatmap analysis was carried out to underline positive and negative relationships between applied treatments and observed traits using the heatmaply package (version 1.5.0) [94].

## 5. Conclusions

This study highlights the considerable potential of the tested consortium of two indigenous soil cyanobacterial strains, *A. cylindrica* and *N. punctiforme*, as promising biofertilizers. These strains exhibited favorable physiological traits, including auxin production, exopolysaccharide secretion, and the synthesis of biologically active compounds such as proteins, sugars, and mineral micronutrients. In greenhouse experiments, soil inoculation with fresh biomass of these cyanobacterial strains applied as a consortium alone or amended with 50% compost or NPK fertilizer led to significant improvements in soil fertility as well as in the growth, biochemical, physiological, and mineral composition of tomato plants. The cyanobacterial consortium also enhanced key tomato agronomic parameters, including fruit productivity, yield, and quality attributes. Ultimately, soil inoculation with the cyanobacterial consortium, whether amended or not, proved more effective and economically advantageous than applying cyanobacterial biomass alone. However, before recommending this bioformulation to farmers, given its potential to reduce chemical fertilizer use by nearly 50%, field trials on tomatoes remain essential to confirm and validate these highly promising results.

## Figures and Tables

**Table 1:** Growth parameters and physiological characteristics of *Nostoc punctiforme* and *Anabaena cylindrica*.

Parameters	*Anabaena cylindrica*	*Nostoc punctiforme*
Growth rates (µ d^−1^)	0.20 ± 0.01 ^a^	0.17 ± 0.01 ^b^
Biomass (g DW L^−1^)	0.77 ± 0.00 ^a^	0.71 ± 0.00 ^b^
Chlorophyll a (mg L^−1^)	3.64 ± 0.59 ^a^	3.08 ± 1.03 ^a^
IAA production (μg mL^−1^)	12.74 ± 0.46 ^a^	4.16 ± 0.25 ^b^
EPS production (mg g^−1^ DW)	42.73 ± 1.40 ^a^	24.67 ± 5.70 ^a^
Nitrogen fixation N_2_	+	+
Phosphate solubilization	+	+

Data are presented as mean ± SE (*n* = 3); means with lowercase letters are statistically different according to Tukey’s HSD test (*p* < 0.05). EPS: exopolysaccharides; DW: dry weight; IAA: auxin; +: presence.

**Table 2:** Biochemical composition of cyanobacterial biomass.

Parameters	*Anabaena cylindrica*	*Nostoc punctiforme*
pH	7.84 ± 0.02 ^a^	7.75 ± 0.02 ^a^
Electrical conductivity (μS cm^−1^)	366 ± 20 ^a^	487 ± 25 ^a^
Phycocyanins (mg L^−1^)	122.5 ± 3.1 ^a^	60.8 ± 2.9 ^b^
Allophycocyanins (mg L^−1^)	60.4 ± 5.4 ^a^	65 ± 4.8 ^a^
Phycoerythrins (mg L^−1^)	4.8 ± 0.8 ^a^	55.4 ± 4.0 ^b^
Polyphenols (µg eq gallic acid mL^−1^)	102.9 ± 0.5 ^a^	58.7 ± 4.9 ^b^
Flavonoids (µg eq catechin mL^−1^)	63.5 ± 0.4 ^a^	22.90 ± 0.14 ^b^
Total carbohydrates (mg g DW^−1^)	32.1 ± 1.7 ^a^	18.8 ± 1.3 ^b^
Proteins (mg g DW^−1^)	53.7 ± 4.8 ^a^	85.1 ± 5.3 ^b^
Total phosphorus (%)	0.12 ± 0.02 ^a^	0.13 ± 0.03 ^a^
Total Kjeldahl nitrogen (%)	2.31 ± 0.02 ^a^	1.98 ± 0.04 ^b^
Total organic carbon (%)	22.22 ± 0.00 ^a^	18.33 ± 0.01 ^b^
K^+^ (%)	0.48 ± 0.01 ^a^	0.47 ± 0.01 ^a^
Ca^2+^ (%)	0.29 ± 0.02 ^a^	0.33 ± 0.02 ^b^
Na^+^ (%)	0.10 ± 0.01 ^a^	0.13 ± 0.01 ^b^

Data are presented as mean ± SE (*n* = 3); means with different lowercase letters are statistically different according to Tukey’s HSD test (*p* < 0.05). Ca^2+^: calcium; K^+^: potassium; Na^+^: sodium; DW: dry weight.

**Table 3:** Physicochemical properties of the soil under different cyanobacterial treatments.

Treatments	pH	EC (mS cm^−1^)	TOC (%)	NTK (mg kg^−1^)	P (mg kg^−1^)	K^+^ (mg kg^−1^)	Na^+^ (mg kg^−1^)	Ca^2+^ (mg kg^−1^)
C-	7.68 ± 0.04 ^a^	0.48 ± 0.03 ^c^	0.70 ± 0.03 ^e^	204.1 ± 5.4 ^d^	147 ± 9 ^d^	939 ± 11 ^d^	247.5 ± 6.4 ^e^	1095.2 ± 2.7 ^d^
C^+^_NPK_	7.15 ± 0.01 ^c^	1.33 ± 0.03 ^a^	0.75 ± 0.06 ^de^	385.6 ± 7.6 ^a^	327 ± 11 ^a^	1336 ± 13 ^a^	317.0 ± 3.9 ^c^	1194 ± 30 ^cd^
C^+^_Comp_	7.23 ± 0.01 ^bc^	1.30 ± 0.01 ^a^	1.93 ± 0.04 ^a^	325 ± 16 ^abc^	385 ± 17 ^a^	1301 ± 5 ^ab^	382.5 ± 3.5 ^a^	1433 ± 21 ^a^
AN	7.35 ± 0.02 ^b^	0.73 ± 0.07 ^b^	1.05 ± 0.03 ^bc^	279.7 ± 7.6 ^bcd^	195 ± 11 ^cd^	1083 ± 31 ^c^	276 ± 11 ^d^	1246 ± 22 ^c^
NO	7.30 ± 0.05 ^bc^	0.82 ± 0.02 ^b^	1.15 ± 0.09 ^bc^	257 ± 21 ^cd^	219 ± 14 ^bcd^	1041 ± 21 ^c^	280.8 ± 9.4 ^d^	1284 ± 21 ^bc^
AN + NO	7.32 ± 0.01 ^b^	0.88 ± 0.03 ^b^	1.18 ± 0.02 ^b^	294.8 ± 5.4 ^bc^	223 ± 10 ^bcd^	1103 ± 21 ^c^	291 ± 11 ^d^	1305 ± 30 ^bc^
AN + NO + 50%NPK	7.22 ± 0.01 ^bc^	1.26 ± 0.04 ^a^	0.93 ± 0.04 ^cd^	363 ± 11 ^ab^	287 ± 36 ^abc^	1282 ± 34 ^ab^	324.5 ± 8.4 ^c^	1227 ± 17 ^c^
AN + NO + 50% Comp	7.27 ± 0.02 ^bc^	1.24 ± 0.04 ^a^	1.75 ± 0.03 ^a^	355.3 ± 7.6 ^ab^	302 ± 37 ^ab^	1228 ± 10 ^b^	345.8 ± 3.7 ^b^	1373 ± 13 ^ab^

Data are presented as mean ± SE (*n* = 3); means with lowercase letters are statistically different according to Tukey’s HSD test (*p* < 0.05). C^−^: negative control; C^+^_NPK_: positive control, full dose of NPK (134 kg N/ha, 56 kg P/ha, and 276 kg K/ha); C^+^_Comp_: positive control, full dose of organic compost (250 g/kg); AN: fresh biomass of *Anabaena cylindrica*; NO: fresh biomass of *Nostoc punctiforme*; AN + NO: consortium of *Anabaena cylindrica* + *Nostoc punctiforme*; AN + NO + 50% NPK: consortium of *Anabaena cylindrica* + *Nostoc punctiforme* + 50% NPK; AN + NO + 50% Comp: consortium of *Anabaena cylindrica* + *Nostoc punctiforme* + 50% compost. TOC: total organic carbon; EC: electrical conductivity; TKN: total Kjeldahl nitrogen; P: Soil P_olsen_; Ca^2+^: calcium; Na^+^: sodium; K^+^: potassium.

**Table 4:** Effect of cyanobacterial treatments on biochemical parameters of tomato fruits.

Treatments	Proteins (mg g^−1^ FW)	Sugars (mg g^−1^ FW)	Polyphenols (mg g^−1^ DW)	Vitamin C (mg 100g^−1^ FW)
C^−^	10.25 ± 0.05 ^a^	32.52 ± 0.01 ^a^	9.00 ± 0.58 ^a^	19.28 ± 0.35 ^a^
C^+^_NPK_	20.43 ± 0.04 ^f^	51.93 ± 0.04 ^ab^	15.33 ± 1.76 ^de^	30.74 ± 0.09 ^bcd^
C^+^_Comp_	23.18 ± 0.02 ^g^	55.02 ± 0.52 ^b^	17.34 ± 1.45 ^e^	32.13 ± 0.84 ^cd^
AN	15.20 ± 0.05 ^b^	35.11 ± 1.02 ^a^	9.67 ± 2.19 ^b^	21.60 ± 0.01 ^ab^
NO	15.39 ± 0.05 ^b^	47.19 ± 0.06 ^b^	11.33 ± 1.20 ^b^	22.63 ± 0.55 ^ab^
AN + NO	16.96 ± 0.01 ^c^	48.75 ± 0.37 ^b^	12.35 ± 1.67 ^ab^	25.42 ± 0.33 ^abc^
AN + NO + 50% NPK	18.41 ± 0.02 ^d^	49.45 ± 0.97 ^ab^	16.33 ± 1.20 ^c^	28.85 ± 0.18 ^cd^
AN + NO + 50% Comp	19.26 ± 0.05 ^e^	50.57 ± 2.00 ^ab^	18.32 ± 1.22 ^c^	30.40 ± 0.36 ^d^

Data are presented as mean ± SE (*n* = 3); means with different lowercase letters are statistically different according to Tukey’s HSD test (*p* < 0.05). FW: fresh weight; DW: dry weight; C^−^: negative control; C^+^_NPK_: positive control, full dose of NPK (134 kg N/ha, 56 kg P/ha, and 276 kg K/ha); C^+^_Comp_: positive control, full dose of organic compost (250 g/kg); AN: Fresh biomass of *Anabaena cylindrica*; NO: fresh biomass of *Nostoc punctiforme*; AN + NO: consortium of *Anabaena cylindrica* + *Nostoc punctiforme*; AN + NO + 50% NPK: consortium of *Anabaena cylindrica* + *Nostoc punctiforme* + 50% NPK; AN + NO + 50% Comp: consortium of *Anabaena cylindrica* + *Nostoc punctiforme* + 50% compost.

**Table 5:** Effect of cyanobacterial treatments on physicochemical parameters of tomato fruits.

Treatments	pH	Total Titratable Acidity (%)	Dry Matter (%)	P (mg g^−1^)	K^+^ (mg g^−1^)	Na^+^ (mg g^−1^)	Ca^2+^ (mg g^−1^)
C^−^	4.25 ± 0.00 ^a^	0.43 ± 0.01 ^d^	5.21 ± 0.02 ^a^	2.21 ± 0.02 ^a^	3.11 ± 0.03 ^a^	0.78 ± 0.01 ^d^	1.74 ± 0.02 ^a^
C^+^_NPK_	4.29 ± 0.02 ^a^	0.34 ± 0.00 ^ab^	6.31 ± 0.04 ^c^	3.39 ± 0.02 ^d^	4.00 ± 0.09 ^bcd^	0.68 ± 0.00 ^bc^	2.84 ± 0.02 ^d^
C^+^_Comp_	4.28 ± 0.04 ^a^	0.35 ± 0.00 ^ab^	6.18 ± 0.05 ^c^	3.77 ± 0.01 ^e^	4.24 ± 0.07 ^cde^	0.67 ± 0.02 ^ab^	2.86 ± 0.02 ^d^
AN	4.26 ± 0.02 ^a^	0.36 ± 0.00 ^b^	5.58 ± 0.11 ^b^	2.30 ± 0.05 ^a^	3.71 ± 0.03 ^abc^	0.72 ± 0.01 ^c^	1.91 ± 0.03 ^a^
NO	4.25 ± 0.00 ^a^	0.42 ± 0.00 ^cd^	5.56 ± 0.07 ^b^	2.62 ± 0.05 ^b^	3.53 ± 0.18 ^ab^	0.67 ± 0.01 ^ab^	2.08 ± 0.00 ^b^
AN + NO	4.24 ± 0.04 ^a^	0.30 ± 0.00 ^a^	5.39 ± 0.02 ^ab^	2.53 ± 0.00 ^b^	3.60 ± 0.05 ^abc^	0.64 ± 0.00 ^ab^	2.51 ± 0.02 ^c^
AN + NO + 50%NPK	4.28 ± 0.07 ^a^	0.31 ± 0.03 ^a^	6.14 ± 0.04 ^c^	3.32 ± 0.00 ^d^	4.75 ± 0.03 ^e^	0.62 ± 0.00 ^a^	2.92 ± 0.04 ^de^
AN + NO + 50%Com	4.27 ± 0.05 ^a^	0.37 ± 0.02 ^bc^	6.04 ± 0.04 ^c^	3.03 ± 0.00 ^c^	4.60 ± 0.04 ^de^	0.63 ± 0.01 ^ab^	3.06 ± 0.00 ^e^

Data are presented as mean ± SE (*n* = 3); means with different lowercase letters are statistically different according to Tukey’s HSD test (*p* < 0.05). P: phosphorus; K^+^: potassium; Na^+^: sodium; Ca^2+^: calcium. C^−^: negative control; C^+^_NPK_: positive control, full dose of NPK (134 kg N/ha, 56 kg P/ha, and 276 kg K/ha); C^+^_Comp_: positive control, full dose of organic compost (250 g/kg); AN: fresh biomass of *Anabaena cylindrica*; NO: fresh biomass of *Nostoc punctiforme*; AN + NO: consortium of *Anabaena cylindrica* + *Nostoc punctiforme*; AN + NO + 50% NPK: consortium of *Anabaena cylindrica* + *Nostoc punctiforme* + 50% NPK; AN + NO + 50% Comp: consortium of *Anabaena cylindrica* + *Nostoc punctiforme* + 50% compost.

**Table 6:** Soil physicochemical properties before application of treatments. Results are means of three replicates (*n* = 3) ± standard error.

Parameters		Results
Physical analysis	pH	7.82 ± 0.02
	Electrical conductivity	0.37 ± 0.02 (mS cm^−1^)
Mechanical analysis	Clay	49 (%)
	Sand	25.7 (%)
	Silt	25.3 (%)
	Soil type	Clay soil
Chemical analysis	Total organic carbon	0.20 ± 0.04 (%)
	Available phosphorus	104.59 ± 0.213 (mg kg^−1^)
	Total nitrogen	173.88 ± 5.35 (mg kg^−1^)
	Potassium	795.50 ± 1.77 (mg kg^−1^)
	Calcium	939.25 ± 16.44 (mg kg^−1^)
	Sodium	195.00 ± 1.41 (mg kg^−1^)

## Data Availability

Data are available on request to the corresponding author.
